# Can 3D Exoscopy-Assisted Surgery Replace the Traditional Endoscopy in Septoplasty? Analysis of Our Two-Year Experience

**DOI:** 10.3390/jcm14155279

**Published:** 2025-07-25

**Authors:** Luciano Catalfamo, Alessandro Calvo, Samuele Cicchiello, Antonino La Fauci, Francesco Saverio De Ponte, Calogero Scozzaro, Danilo De Rinaldis

**Affiliations:** Department of Biomedical, Dental and Morphological and Functional Imaging (BIOMORF), University of Messina, Via Consolare Valeria n. 1, 98122 Messina, Italy; lcatalfamo@unime.it (L.C.); alessandrcalvo@gmail.com (A.C.); samuele.cicchiello@gmail.com (S.C.); ninnilf21@gmail.com (A.L.F.); lc.lab@hotmail.it (F.S.D.P.); calogerosco94@gmail.com (C.S.)

**Keywords:** exoscopy, new technologies, 3D surgery, magnification, rhinoseptoplasty

## Abstract

**Background/Objectives**: Septoplasty is a commonly performed surgical procedure aimed at correcting nasal septal deviations, to improve nasal airflow and respiratory function. Traditional approaches to septal correction rely on either direct visualization or endoscopic guidance. Recently, a novel technology known as exoscopy has been introduced into surgical practice. Exoscopy is an “advanced magnification system” that provides an enlarged, three-dimensional view of the operating field. In this article, we present our experience with exoscope-assisted septoplasty, developed over the last two years, and compare it with our extensive experience using the endoscopic approach. **Methods**: Our case series includes 26 patients, predominantly males and young adults, who underwent exoscope-assisted septoplasty. We discuss the primary advantages of this technique and, most importantly, provide an analysis of its learning curve. The cohort of patients treated using the exoscopic approach was compared with a control group of 26 patients who underwent endoscope-guided septoplasty, randomly selected from our broader clinical database. Finally, we present a representative surgical case that details all phases of the exoscope-assisted procedure. **Results**: Our surgical experience has demonstrated that exoscopy is a safe and effective tool for performing septoplasty. Moreover, the learning curve associated with this technique exhibits a rapid and progressive improvement. Notably, exoscopy provides a substantial educational benefit for trainees and medical students, as it enables them to share the same visual perspective as the lead surgeon. **Conclusions**: Although further studies are required to validate this approach, we believe that exoscopy represents a promising advancement for a wide range of head and neck procedures, and certainly for septoplasty.

## 1. Introduction

Septoplasty is among the most commonly performed procedures in otolaryngology and maxillofacial surgery, primarily employed to correct nasal septal deviations responsible for airway obstruction [1]. The main objective of the procedure is to restore proper nasal airflow by realigning the nasal septum [2,3]. Traditionally, this procedure is performed without the aid of advanced technical tools, relying instead on direct visualization of the septum and surgical field. However, it is undeniable that visualization of the septum through the nostril is significantly limited, making instrument manipulation within such a confined space particularly challenging. This has long represented one of the most critical challenges in nasal cavity surgery, which is why scientific research in this field has largely focused on improving intraoperative visibility.

Visibility is a fundamental aspect of surgery across all surgical disciplines. Consider, for instance, the advances in abdominal surgery following the introduction of laparoscopic procedures.

In the context of nasal cavity surgery, the first notable improvements in visualization occurred in the 1990s, when French authors reported the use of endoscopic probes in this type of procedure [4]. Prior to that, endoscopy had been used primarily in paranasal sinus surgery, where its application dates back to the 1950s [5,6].

Endoscopy is a technique that enables direct visualization of a surgical site through the use of an optical probe. In essence, it allows the surgeon’s vision to be projected to the tip of the probe as if the eyes were located there. The main advantage of endoscopy lies in its ability to provide access to anatomical areas that would otherwise be unreachable without the probe. However, its main limitation concerns the restricted field of view, which is confined to a circular area whose diameter is proportional to that of the probe. Additionally, visibility may often be impaired when the tip of the probe becomes fogged. Nevertheless, endoscopy remains the only technique—apart from direct visualization—currently employed in nasal cavity surgery, as it is the sole method capable of assisting the surgeon in such procedures [7].

However, a recent advancement known as exoscopy has been introduced into surgical practice. First described in 1994 as an evolution of microscopy [8], exoscopy represents an “advanced magnification system” similar to traditional microscopy, but with the significant distinction of providing a high-definition three-dimensional view. This 3D image is displayed on a 4K monitor and can be directly observed using specialized polarized glasses worn during the procedure.

Exoscopy has recently been described in parotid gland surgery [9], although its use is particularly widespread in neurosurgery, where it has been associated with favorable outcomes in the resection of pineal region tumors via an infratentorial approach [10,11,12,13], in neurovascular pathology [14], and in the treatment of olfactory neuroblastoma [15]. Meanwhile, longer operative times have been reported for spinal surgery assisted by exoscopy compared to conventional microsurgery [16]. The placement of a stent for carotid artery stenosis via exoscopy has also been documented [17]. Exoscopy has additionally been employed in pediatric surgery [18].

In recent years, exoscopy has gained increasing attention in the field of otorhinolaryngology. Its application has been explored in various subspecialties, including microlaryngeal surgery, middle ear procedures, transoral pharyngeal resections, and even thyroid surgery. Several studies have highlighted the benefits of 3D visualization, improved ergonomics, and the ability to share the surgical field with trainees and assistants through external monitors [19]. In particular, the use of exoscopy in microlaryngeal procedures has demonstrated comparable outcomes to those achieved with conventional microscopy, with the added advantage of an expanded depth of field and enhanced surgeon comfort [20]. In otologic surgery, exoscopy has been reported as a viable alternative to the operating microscope, especially in tympanoplasty and mastoidectomy, although it remains limited by the need for optimal lighting and line-of-sight [21].

Despite these promising results, evidence regarding the use of exoscopy in nasal surgery remains scarce. To the best of our knowledge, no published study has yet addressed the application of exoscopy in septoplasty. The present work therefore aims to fill this gap by providing a detailed account of our initial experience with this technique in nasal septum surgery.

The loss of stereopsis and the requirement for a high level of hand–eye coordination represent challenges to be overcome when performing exoscopic procedures; however, both are expected to improve with training [22,23].

In this article, we present our experience with septoplasty performed via exoscopy, highlighting the advantages of this approach. We provided a detailed description of the surgical technique and compare it with our previous experience using endoscopy.

## 2. How the Exoscope Works

**Figure 1:**
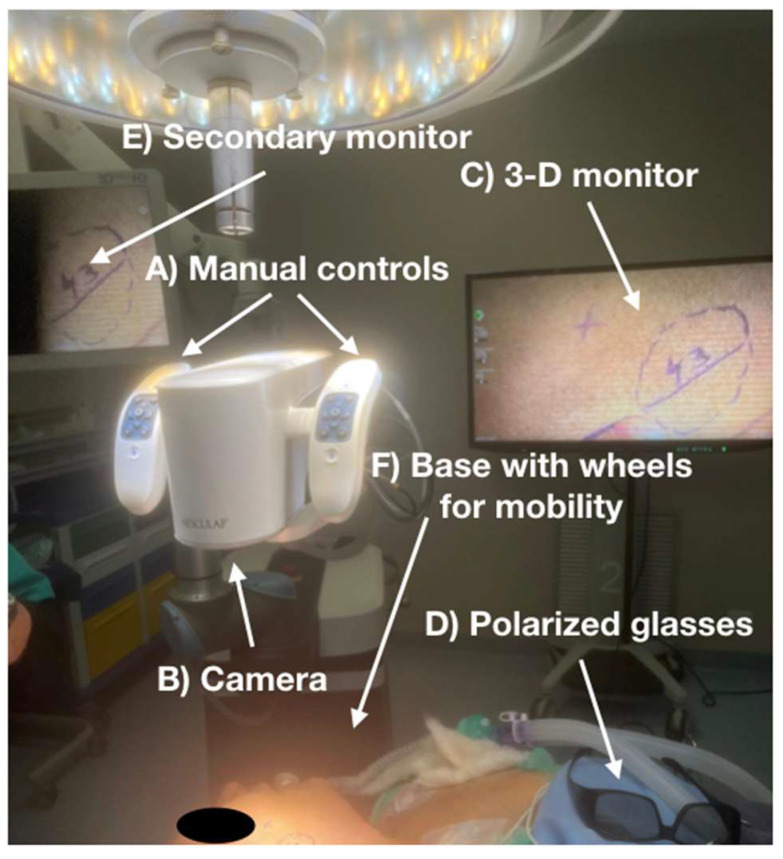
Components of the Aesculap Aeos^®^ Exoscope System (B. Braun Group, Italy): (**A**) Central robotic arm with dual joysticks; (**B**) 3D 4K optical camera head; (**C**) Primary monitor (32″); (**D**) Polarized glasses for 3D visualization; (**E**) Additional OR monitors; (**F**) Base with wheels for mobility.

The exoscope (Figure 1) is a relatively large instrument composed of a robotic arm equipped with dual joysticks and a central lens that functions as a camera. The right and left joysticks, together with the central camera, form a single integrated unit. The device is operated by gripping both joysticks. Each joystick is fitted with a lever that, when engaged, allows free movement, whereas when disengaged, it restricts movement, keeping the unit completely stationary.

This allows the camera to focus on a specific area of the surgical field, which is then “magnified”, that is, captured by the camera and displayed on the monitor in an enlarged (8–30×) and three-dimensional view. The monitor is connected to the exoscope via another robotic arm, as shown in Figure 1. However, multiple monitors can be connected to the device using linking cables.

This represent a significant advancement in surgery, as the entire operating room staff can follow the procedure from the same visual perspective as the lead surgeon. In fact, the exoscope can be connect to two additional monitors (in addition to the one used by the primary surgeon) positioned at various locations within the operating room, allowing all personnel present to observe the procedure from the surgeon’s point of view. Naturally, observers must also wear the same polarized glasses worn by the operating team (Figure 1). In order to view the surgical field in 3D, it is essential that the observer be positioned directly in front of the monitor. This requirement applies to both operators and observers, which is why multiple monitors are positioned throughout the operating room.

The exoscope camera is equipped with an autofocus function, which greatly simplifies the operator’s work when the camera needs to be repositioned or the operating field changed. The camera does not interfere with the surgeon’s maneuvers; its sole purpose is to magnify the surgical field, which the surgeon observes directly on the 3D monitor while their hands remain actively engaged within the operative area.

The exoscope, including its monitors, is equipped with wheels, making each component mobile and allowing for quick transportation into the operating room. However, the overall volume of the device is not considerable; therefore, it is generally positioned in the operating room the day before the scheduled procedure. In other words, the placement of the exoscope does not extend the overall duration of the surgical procedure.

The exoscope system used in our study was the Aesculap Aeos^®^ (produced by B. Braun Group, Italy), a high-definition three-dimensional magnification system designed for surgical visualization. The setup is composed of several key elements, as illustrated in Figure 1, each of which will be detailed below to ensure full procedural reproducibility.

(1) **Central Robotic Arm and Optical Unit**
  The heart of the system is a robotic arm mounted on a mobile base with integrated wheels for easy maneuverability. The arm supports a high-resolution 4K 3D digital camera equipped with an autofocus system and adjustable zoom (8–30x magnification). The camera is controlled via dual joysticks, also mounted on the arm, allowing precise movement and locking of the optical unit.
  The camera head contains two optical channels to generate stereoscopic images and is encased in a lightweight carbon fiber housing.
(2) **Visualization System (Monitor Setup)**
  The visual output is displayed on a 32-inch 4K 3D monitor, which is mounted on a secondary articulated arm and can be freely positioned in the operating room. The monitor provides real-time stereoscopic visualization via passive polarized 3D glasses, which must be worn by all observers and members of the surgical team. To facilitate education and team coordination, two additional monitors were connected using HDMI cables and placed at strategic positions within the OR. All monitors must be aligned perpendicular to the viewer’s line-of-sight to maintain proper stereopsis.
(3) **Illumination and Image Optimization**
  The system uses LED-based cold light sources integrated into the optical head, providing shadow-free and homogenous illumination. The brightness and contrast can be adjusted manually or via automatic presets, depending on the area of interest. An integrated anti-fogging mechanism minimizes condensation on the lens during prolonged procedures.
(4) **Operating Room Integration**
  The entire setup (robotic arm, optical unit, and monitor) is pre-positioned the day before surgery to minimize operative delays. All components are modular and mobile, requiring only standard power outlets and HDMI video connections. The exoscope does not require sterilization of the optical head, as it remains outside the sterile field. Instead, the joysticks and handle controls are sterilizable or covered with sterile drapes.
(5) **Software Interface**
  The system’s software allows for the following:
  - Live recording of the procedure (MP4 format, up to 4K resolution);
  - Still image capture for documentation;
  - Real-time digital zoom and panning;
  - On-screen annotation tools during teaching sessions.
(6) **Training and Ergonomics**
  The main operator observes the monitor while performing the surgery, which requires a high degree of hand–eye coordination. During our study, the learning curve was evaluated by tracking operative times (see Table 1). Operator comfort was significantly improved compared to endoscopy, as the posture remains neutral and the monitor position can be freely adjusted.

## 3. Surgical Anatomy of the Nasal Septum and Septoplasty

The nasal septum is the primary structural component of the nose. It is an internal structure that divides the two sides of the nasal cavity and provides support to the nasal tip and columella [24].

**Figure 2:**
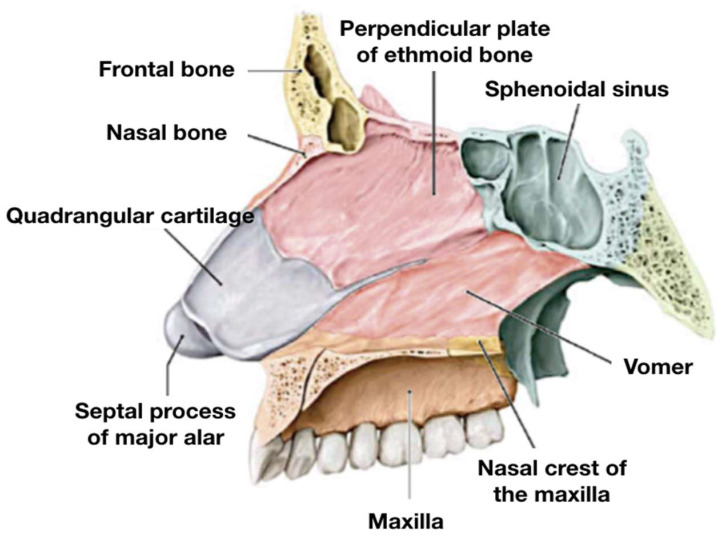
Anatomy of nasal septum: the quadrangular cartilage and the septal process of major alar cartilage constitute the cartilaginous portion; the PPE and vomer form the bony component; the nasal crest of the maxilla is clearly visible, articulating with the inferior margin of the vomer, with the “tail” of the quadrangular cartilage interposed between the two bones.

The nasal septum (Figure 2) is composed of three main components: membranous, cartilaginous, and bony [25,26]. The membranous portion, located in the nasal vestibule, consists of fibrous tissue and lies between the medial crura of the two major alar cartilages.

The quadrangular cartilage fits between the two bones that comprise the bony part of the septum; it represents the most anterior and inferior part of the nasal septum and lies between the medial crura of the two major alar cartilages.

The cartilaginous portion consists of the “quadrangular cartilage” and the septal part of the major alar cartilages. The quadrangular cartilage fits precisely between the two bones that form the bony part of the nasal septum: the perpendicular plate of the ethmoid (PPE) superiorly and the vomer inferiorly. These bones are fused posteriorly, while anteriorly, they are separated by the “tail” of the quadrangular cartilage. Inferiorly, the vomer articulates with the nasal crest of the maxilla and anteriorly with the anterior nasal spine.

From a vascular standpoint, the most relevant area is the anterior–inferior portion of the septum, where the “Kiesselbach’s plexus” (also known as “Little’s area”) is located. This highly vascularized region is the site of convergence for branches of both the internal and external carotid systems and is the most frequent source of anterior epistaxis. During septoplasty, special care must be taken to avoid excessive trauma in this region to minimize bleeding risks.

Regarding preoperative CT imaging and intraoperative navigation, key anatomical landmarks include the anterior nasal spine, bony-cartilaginous junction, nasal floor midline, and the junction between the vomer and the PPE. These structures help in orientation during flap elevation, septal mobilization, and correction of posterior deviations. Intraoperatively, the anterior nasal spine serves as a reliable landmark for re-centering the caudal septum, while the nasal floor midline aids in aligning the inferior septal border.

Three main surgical approaches are generally employed to perform septoplasty: closed, endoscopic, and open techniques [27]. The procedure typically involves exposing the cartilaginous septum via a submucoperichondrial flap. Once exposed, the cartilaginous component is mobilized, repositioned, or resected and then reconstructed or stabilized [28]. In cases where the bony septum is involved, the exposure must be extended posteriorly, allowing reshaping of the deviated bone using instruments such as Killian’s forceps.

Septoplasty may be performed via either a closed or open approach; in the latter case, it is often combined with rhinoplasty [29].

## 4. Our Experience

In this article, we share our experience with the use of exoscopy in septoplasty procedures. Our previous expertise was primarily rooted in a close-up approach to the septum using traditional techniques under endoscopic visualization, an experience that began over fifteen years ago and encompasses more than 2250 Septoplasty cases. When exoscopy was proposed as a replacement for Endoscopy, our initial reaction was one of hesitation. We were aware that it would not allow for the insertion of a scope into the nasal cavity and that visualization would remain extra-situ, detached from the surgical field. Nevertheless, we were intrigued by the possibility that the 3D magnification of the septum provided by exoscpopy might enhance surgical precision. This curiosity ultimately led us to explore its potential. Our experience with exoscopy began two years ago (we have been using it since February 2022) and our current case series includes over 30 procedures performed with this technique, although only 26 of these have been adequately documented. For comparison purposes, we collected data from an equal number of 26 patients treated between 2020 and 2022 using endoscopic visualization.

Patients in the control group (endoscopic septoplasty) were retrospectively selected from our institutional surgical database. Inclusion criteria for both groups were: (1) age between 18 and 65 years, (2) documented nasal obstruction due to septal deviation, and (3) no history of major sinonasal surgery. Exclusion criteria included presence of nasal polyposis, concurrent sinus surgery, or craniofacial malformations. For the control group, we excluded patients with incomplete medical records or follow-up data. Patients were matched by age range, gender distribution, and side and cause of deviation to minimize selection bias. No randomization was performed, as this was a retrospective, non-randomized comparative study.

The two patients groups (endoscopic vs. exoscopic) were compared in terms of complications, and resolution—or lack thereof—of obstructive symptoms following treatment.

Our exoscopic case series is presented in Table 1. It includes 26 young adult patients (aged between 19 and 56 y.o.), comprising 18 males and 8 females, with 14 cases of rightward septal deviation and 12 of leftward deviation.

All patients underwent the procedure under General Anesthesia; however, the same technique would be feasible under Local Anesthesia with Sedation.

In most cases, the septal deviation was attributed to a previous traumatic event; only five patients underwent Septoplasty to correct damage related to drug use (specifically, cocaine abuse). For three patients, information regarding the cause of the deviation was unavailable—in this instances an unrecognized traumatic event is suspected). Few complications were recorded, all of which were minor. Specifically, we observed three cases of intraoperative bleeding, two cases of postoperative bleeding (during nasal packing removal), and two cases of postoperative infections caused by opportunistic pathogens. In the majority of cases, the procedure was successful, meaning that patients no longer experienced the same respiratory difficulties reported prior to surgery; persistent symptoms were noted only in five cases.

Finally, the last column of Table 1 presents the operative times for each procedure, which reveal a clear downward trend. After two years of experience with exoscopic procedures, operative times have decreased by an average of 35%, indicating a significant learning curve. The procedures were performed by only two surgeons, who alternated between cases; however, given the overlap in their surgical experience, their performance can be considered equivalent.

Operative times were measured from the beginning of the procedure (initial incision, following patient intubation) to the end of the intervention (prior to patient emergence from anesthesia). In other words, the recorded operative times refer exclusively to the surgical procedure itself.

Table 2 presents 26 endoscopic cases drawn from our previous clinical case series. All patients underwent endoscopic Septoplasty between 2020 and 2022. A comparable incidence of postoperative complications can be observed in both groups, while a slightly higher incidence of intraoperative complications was reported in the exoscopic group (3 in the exoscopic group vs. 1 in the endoscopic group). As with the exoscopic group, the majority of patients in the endoscopic group experienced resolution of obstructive symptoms following treatment (80.7% exoscopic group vs. 84.6% endoscopic group).

**Figure 3:**
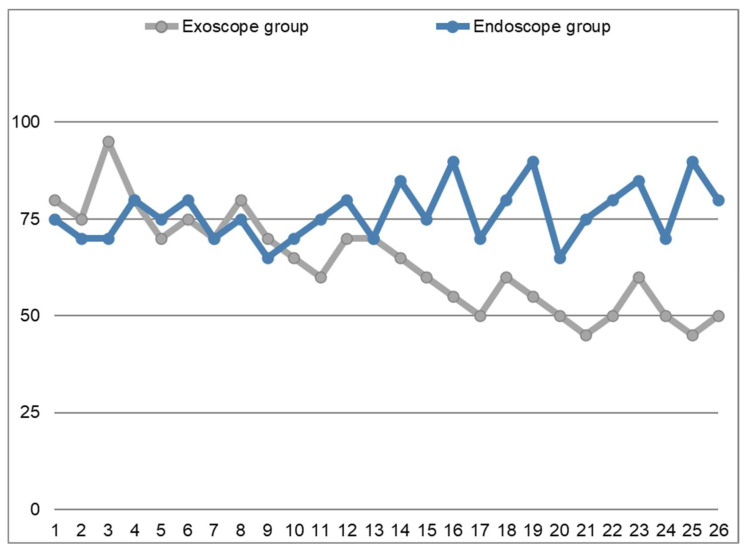
The x-axis represents the patients from the two case series, while the y-axis indicates operative times expressed in minutes; in the endoscopic group (blue line) operative times remain stable, initially slightly higher than those in the exoscopic group (grey line); however, following a period of partial overlap, operative times in exoscopic group ultimately became shorter than those in the endoscopic group; the grey line corresponds to the learning curve of the exoscope-guided septoplasty procedure. **Operative Time:** Duration of the procedure in minutes.

However, from our perspective, the most noteworthy finding concerns the differences in operative times observed between the two groups. These data are illustrated in the graph shown in Figure 3, which demonstrates that the surgical times in the endoscopic group (blue line) remain stable, fluctuating slightly above and below the line of the 75-min-mark, which represents the average time required to perform this procedure in our experience. In contrast, the reference line representing the exoscopic group (grey line) reveals that operative times were initially longer compared to the endoscopic group. However, following a period of partial overlap, operative times eventually become consistently shorter. The gray line corresponds to the Learning Curve of the Exoscope-guided Septoplasty.

A statistical comparison between the operative times of the endoscopic and exoscopic groups was conducted using an independent *t*-test. The results revealed the following:

- For the endoscopic group, the mean operative time was 72.69 min (SD = 8.93);
- For the exoscopic group, the mean operative time was 74.23 min (SD = 8.55);
- The *t*-test yielded a *t*-statistic of −1.25 and a *p*-value of 0.22.

This indicates that there was no statistically significant difference between the two groups in terms of operative times. However, it is important to note that the trend over time shows a decreasing duration of the procedures in the exoscopic group as the surgeons gained more experience with the new technology.

Despite the absence of a significant difference in operative times, it is worth highlighting that the exoscopic approach allowed for a better 3D visualization of the surgical field, which might have contributed to an improved understanding of anatomy, better precision in surgical maneuvers, and a more efficient learning curve for the surgeons.

In summary, although the exoscopic method did not show a statistically significant reduction in operative time compared to the endoscopic approach, the learning curve was clearly evident, with a reduction in operative times over time. Moreover, the 3D magnification and better visualization of the surgical field may provide other advantages that were not captured solely by the operative time comparison. This suggests that, with further experience, exoscopic procedures may become more efficient, with the potential to further reduce operative times and improve surgical outcomes.

## 5. Clinical Case

Below is an illustrative clinical case (case n° 12 in Table 1). Unfortunately, it is not possible to replicate in the following images the same three-dimensional visual experience perceived by the surgeon and others present in the operating room, as doing so would require specific polarized glasses.

The case is a 29-year-old woman with chronic nasal obstruction resulting from a previous car accident, presenting with a rightwards nasal septal deviation and reporting breathing difficulties, particularly at night. Physical examination combined with computed tomography (CT) scan revealed a pronounced deviation of the nasal septum towards the right side with a prominent posterior bony spur.

**Figure 4:**
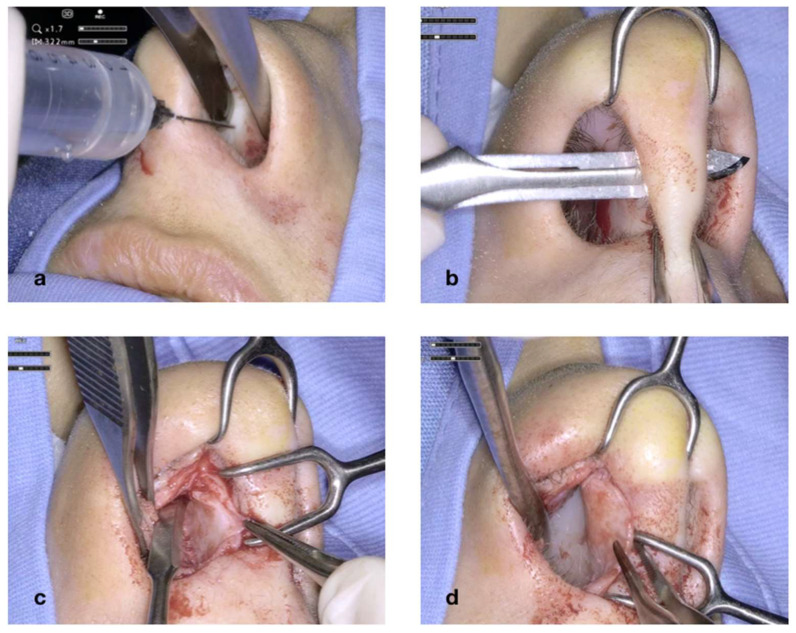
Infiltration of the nasal mucosa with Mepivacaine and Adrenaline (1:100,0000) (**a**); transcolumellar incision (**b**); exposure of the cartilaginous septum (**c**,**d**).

Figure 4 illustrates the initial steps of the procedure: The nasal mucosa overlying the cartilaginous septum is bilaterally infiltrated with Mepivacaine and Adrenaline (1:100.000) (Figure 4a). A transcolumellar incision is performed with a #15 blade through the fibrous septum (Figure 4b). The cartilaginous septum is well exposed on both sides (Figure 4c,d). The transcolumellar incision is not an additional access required for the use of the exoscope, but rather, a component of our standard septoplasty technique.

**Figure 5:**
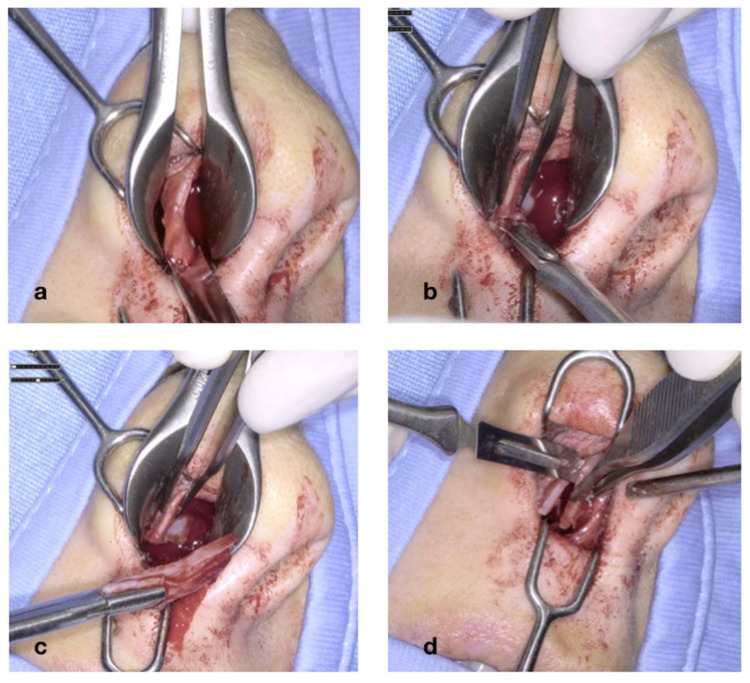
The cartilaginous septum is detached from the ANS and the midline of the nasal floor (**a**); its inferior and anterior portions are trimmed with a #15 scalpel blade and excised (**b**–**d**).

In Figure 5, the cartilaginous portion of the septum is freed from the bony adhesions. The septum is detached from the anterior nasal spine, while its inferior border is released from the midline of the nasal floor (Figure 5a); once the septum is detached, its inferior and anterior parts are trimmed with the scalpel blade and then excised (Figure 5b,d).

**Figure 6:**
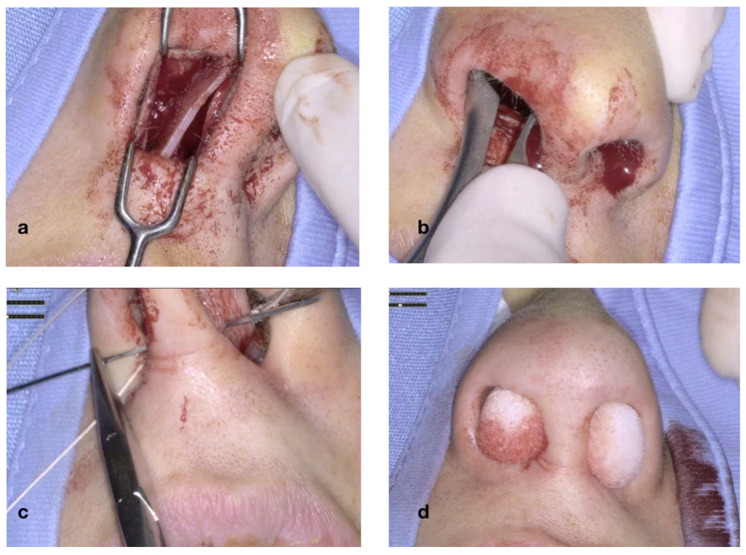
Fully mobilized cartilaginous septum (**a**); correction of the septal deviation using Killian’s forceps (**b**); through and through suture with knot on the opposite side of the deviation (**c**); placement of nasal packing (**d**).

The cartilaginous septum is now fully mobilized (Figure 6a), as it remains attached to the bony septum (vomer and PPE) only superiorly and posteriorly. The deviation of the bony portion of the septum is then corrected using a Killian’s forceps, with the prongs inserted through the nostril and advanced upwards until they contact with the bony septum (Figure 6b). The prongs are then opened; this maneuver must be performed on both sides, regardless of the side of the deviation, as correcting only the deviated side may lead to overcorrection. Finally, the last stage of septoplasty involves securing the cartilaginous septum with a through–through suture (Figure 6c). It is crucial that the knot be tied on the side opposite of the initial deviation, so that the suture exerts tension counteracting the elastic memory of the tissues. The procedure concludes with the placement of nasal packing, which is removed within 3–4 days (Figure 6d).

In this particular case, no complications—either intra- or postoperative—were observed; the procedure lasted 70 min and had a successful outcome for the patient.

In Table 3, the main technical and practical characteristics of the two types of procedures under comparison are presented.

## 6. Discussion

In recent years, new technologies have increasingly permeated various medical disciplines, with maxillofacial surgery emerging as one of the most dynamic and innovative fields in this regard. Technological advancements such as artificial intelligence (AI), robotic surgery, image-guided navigation, and augmented reality (AR) have rapidly transitioned from theoretical concepts to practical tools integrated into routine surgical workflows. These innovations have not only enhanced procedural accuracy, operational efficiency, and patient safety but also facilitated the development of highly personalized treatment strategies, real-time intraoperative guidance, and improved clinical outcomes.

While these technologies were once considered distant aspirations, today they have become a tangible reality, revolutionizing medical practices across the globe. The adoption of such cutting-edge technologies promises to improve the overall quality of care, reduce the risk of complications, and enable more precise and less invasive interventions. However, the introduction of these technologies often requires time for their full potential to be realized, and early-stage use can sometimes be associated with a learning curve that impacts initial outcomes.

The use of exoscopy in septoplasty, as presented in this study, exemplifies a significant technological advancement in this domain. Compared to traditional endoscopic septoplasty, exoscopy offers several advantages, including stereoscopic 3D visualization, a high-definition field of view, and improved ergonomic comfort for the surgeon. These factors contribute to an enhanced ability to identify and correct anatomical deviations with greater precision and reduced physical strain during prolonged procedures. However, the technology’s inherent cost remains a primary limitation, with many surgical centers finding the acquisition of exoscopic systems financially prohibitive. Despite this, exoscopy represents an important step forward in surgical technology and, as such, warrants further exploration and adoption in specialized centers.

- **Comparison with Alternative Approaches**

In the context of septoplasty, there are several alternative techniques that are commonly employed, each with its own advantages and limitations. The traditional closed septoplasty, which involves manual manipulation and limited visualization, has long been considered the gold standard. However, this technique is associated with challenges in visualizing and correcting complex deviations, particularly in the posterior or superior portions of the septum. While endoscopic septoplasty has gained traction as an improvement over traditional methods due to its ability to provide better visualization, particularly through the use of high-definition endoscopes, it still suffers from limitations related to depth perception and field of view. The confined nature of endoscopic visualization can make it difficult to assess and correct deviations, especially in challenging cases.

In contrast, exoscopic septoplasty provides superior three-dimensional visualization, offering an improved view of both the anterior and posterior aspects of the nasal septum. The 3D magnification provided by the exoscope enhances the surgeon’s ability to identify subtle deviations and ensure precise corrections. Moreover, the ability to visualize the entire surgical field without the need for direct line-of-sight provides ergonomic advantages that may reduce surgeon fatigue and improve performance during lengthy procedures.

While microsurgical techniques have also been employed in septoplasty, they often require significant expertise in maneuvering fine instruments under magnification, which can be challenging in certain anatomies. Exoscopy, with its superior optical resolution, allows for better visualization and greater maneuverability, potentially reducing the risk of iatrogenic injury and improving overall surgical outcomes.

- **Challenges and Learning Curve**

The introduction of any new technology is often accompanied by a learning curve, and the use of exoscopy in septoplasty is no exception. While the technology offers numerous advantages, initial use may lead to increased intraoperative bleeding and slightly longer operative times. However, as the surgeon becomes more proficient in the use of the exoscope, these issues tend to diminish. In our study, we observed a mild increase in intraoperative bleeding in the first year of exoscope use, compared to a decrease in the second year, which supports the notion that surgeon skill improves over time with consistent practice and experience.

This improvement over time is consistent with the findings of other studies in the literature, which have shown that surgeon familiarity with new technologies significantly enhances both operational efficiency and clinical outcomes. It is important to note that while intraoperative bleeding was observed in some cases, it was mild and did not lead to any significant clinical complications or adverse outcomes. Nonetheless, this remains an area for potential optimization as the technology evolves.

Technological advancements often enhance surgical procedures, reduce the risk of complications, improve patient outcomes, and streamline the work of healthcare professionals from the outset; however, they may sometimes require time to clearly demonstrate their full potential. In our experience, as presented in this article, the use of an exoscope in septoplasty demonstrates a comparable incidence of postoperative complications and unresolved cases following the procedure when compared to endoscopic septoplasty. Conversely, a slight increase in intraoperative bleeding was observed in the exoscope group; however, this complication occurred more frequently during the first year of exoscope use compared to the second year (2 cases vs. 1). This finding supports the notion that the surgeon’s ability to maneuver the device improves significantly over a relatively short period of time, as confirmed by the international literature [20,30], although the number of publications on the topic remains limited, with none specifically addressing septoplasty. In any case, the hemorrhagic episodes were mild and had no clinical relevance.

- **Exoscopy in Training and Education**

One of the most promising aspects of exoscopic technology is its potential to revolutionize surgical training. Traditional surgical education often involves a passive observation of procedures, with trainees relying on 2D visualizations and limited exposure to real-time operative perspectives. Exoscopy, however, offers a stereoscopic 3D view of the surgical field, allowing trainees and students to experience the procedure from the perspective of the lead surgeon. This capability enhances the understanding of complex anatomical relationships and surgical steps, significantly improving the learning experience. Furthermore, the ability to interactively engage with the surgical field in a manner that mimics the surgeon’s viewpoint allows for active learning in a way that traditional methods cannot replicate.

Exoscopy thus has the potential to play a pivotal role in the education of future surgeons, providing an innovative tool to bridge the gap between theory and practice. This could lead to better-prepared surgeons and potentially fewer errors in the early stages of their careers.

Looking ahead, the integration of artificial intelligence (AI) and augmented reality (AR) into exoscopy could offer further advancements. AI could potentially assist in preoperative planning, intraoperative guidance, and decision-making by analyzing pre-surgical imaging and suggesting optimal surgical approaches. Meanwhile, AR could overlay virtual anatomical models onto the real surgical field, further enhancing visualization and guiding the surgeon in real time. These technological developments could ultimately streamline the surgical process and further improve patient outcomes.

## 7. Limitations

### 7.1. Retrospective Study Design

A key limitation of this study is its retrospective design, which inherently restricts the ability to establish causality and limits the control over confounding variables. Retrospective studies are prone to selection bias and often lack the rigorous data collection protocols present in prospective studies, potentially affecting the validity and reliability of the findings.

### 7.2. Lack of Standardized Scoring Systems

Another important limitation is the absence of standardized, validated scoring systems, such as the NOSE (Nasal Obstruction Symptom Evaluation) score, for objectively assessing functional outcomes. While patient-reported symptomatic resolution was used to evaluate treatment success, this subjective measure does not provide the quantitative, validated data that a scoring system could offer. The integration of such scoring systems into clinical practice would allow for more robust and comparable outcomes across studies.

### 7.3. Small Sample Size and Descriptive Study Design

The relatively small sample size used in this study limits the generalizability and statistical power of the results. Given the exploratory and descriptive nature of this study, the findings may not be applicable to a broader population or more diverse clinical settings. Larger, prospective studies with more participants would be essential for confirming the observed trends and improving the robustness of the conclusions.

### 7.4. Short-Term Follow-Up

Another limitation is the short duration of follow-up for the patients in this study. Without long-term follow-up data, it is difficult to assess the durability of the surgical outcomes or identify potential late complications. Further studies with longer follow-up periods would provide crucial information regarding the longevity of the improvements achieved through exoscopic-assisted septoplasty.

### 7.5. Lack of Comparison with Alternative Non-Conventional Approaches

While this study provides a comparison between exoscopic-assisted septoplasty and traditional endoscopic methods, it does not explore or compare alternative, non-conventional techniques such as laser-assisted septoplasty or minimally invasive approaches. Including these comparisons could offer a more complete picture of the potential advantages and disadvantages of exoscopy within the broader spectrum of septoplasty techniques.

### 7.6. Surgeon Experience and Variability

The role of surgeon experience is another limitation not fully addressed in this study. The ability to effectively use the exoscope can vary widely depending on the individual surgeon’s experience and familiarity with the device. This could influence the consistency of the results and may explain some of the variability in the observed outcomes, especially during the initial phases of exoscope use. As proficiency improves with experience, this limitation may diminish over time.

### 7.7. Potential Bias in Patient Selection

Given the retrospective nature of this study, there may have been a selection bias in the patient cohort, which could affect the generalizability of the findings. Patients who were selected for exoscope-assisted septoplasty may have differed in terms of clinical characteristics or severity of nasal obstruction compared to those undergoing traditional methods. Future prospective studies with randomized patient allocation would help minimize this type of bias and provide more reliable data.

### 7.8. Justification for Limitations

It is important to note that this study is a pilot investigation, and the limitations outlined above are inherent to studies of this nature. Given the preliminary scope of this work, the findings provide a foundational understanding of exoscopic-assisted septoplasty, but they must be interpreted with caution. The limitations of this study, including the retrospective design, small sample size, and lack of standardized outcome measures, are typical of pilot studies. Future, larger-scale prospective studies with more rigorous methodologies will be necessary to validate these results, address the current limitations, and further elucidate the potential benefits of exoscopic-assisted septoplasty.

## 8. Conclusions

Exoscopy, although still in its early stages of adoption, holds significant promise for enhancing surgical outcomes across a range of medical specialties, with maxillofacial surgery being a key area of application. As demonstrated in this pilot study, exoscopy offers notable improvements in precision and visualization during surgical procedures, which may lead to better patient outcomes, enhanced safety, and more efficient workflows.

However, it is important to recognize that the technology’s primary limitation remains its cost. The financial investment required to acquire and implement an exoscopic system may be prohibitive for many healthcare facilities, particularly those with limited resources. This presents a challenge to its widespread adoption, especially in resource-constrained environments.

Despite these challenges, exoscopy represents a clear example of the potential benefits of incorporating innovative technologies into clinical practice. As with any cutting-edge advancement, the initial cost barrier may be overcome over time as the technology matures, becomes more accessible, and demonstrates its long-term value in clinical settings. For forward-thinking medical centers, investing in exoscopic systems may not only improve surgical practice but also contribute to a more efficient, future-ready healthcare system.

This study is a pilot investigation, and although the preliminary results are promising, further research is required to confirm and expand upon these findings. Larger-scale, multi-center studies with more robust sample sizes and standardized outcome measures are needed to establish the full clinical efficacy and long-term benefits of exoscopy in maxillofacial surgery.

In conclusion, while exoscopy is still a developing technology, its integration into surgical practice holds considerable promise for transforming the way we approach surgeries, particularly in the field of maxillofacial surgery. Continued research, improved access, and greater familiarity with the technology will likely pave the way for broader adoption, ultimately benefiting both surgeons and patients alike.

## Figures and Tables

**Table 1:** Exoscope group.

Patient	Sex	Age	Side *	Cause **	Complications ^§^	Outcomes ^§§^	Operative Time
1	M	32	L	T	NO	Resolutive	80′
2	M	24	L	T	NO	Resolutive	75′
3	W	43	R	D	Intra-op	Not Res.	94′
4	M	25	R	Unknown	NO	Resolutive	80′
5	M	54	R	T	NO	Resolutive	70′
6	M	34	L	T	Post-op	Resolutive	75′
7	W	27	R	T	NO	Not Res.	70′
8	W	32	L	D	NO	Resolutive	80′
9	M	19	L	T	Intra-op	Resolutive	70′
10	M	47	R	D	NO	Resolutive	65′
11	M	36	L	Unknown	NO	Not Res.	60′
12	W	29	R	T	NO	Resolutive	70′
13	M	25	R	T	Post-op	Resolutive	70′
14	M	52	R	T	NO	Resolutive	65′
15	W	37	L	T	NO	Not Res.	60′
16	W	33	L	T	Intra-op	Resolutive	55′
17	M	27	R	D	NO	Resolutive	50′
18	M	31	L	T	NO	Resolutive	60′
19	M	42	R	T	Post-op	Resolutive	55′
20	M	20	R	Unknown	NO	Resolutive	50′
21	W	56	R	T	NO	Resolutive	45′
22	M	44	L	D	NO	Not Res.	50′
23	W	35	R	T	NO	Resolutive	60′
24	M	31	L	T	Post-op	Resolutive	50′
25	M	25	L	T	NO	Resolutive	45′
26	M	37	R	T	NO	Resolutive	50′

* Side of deviation: L (Left), R (Right); ** Cause: T (post-Traumatic deviation), D (Drugs); ^§^ Complications: Intraoperative (bleeding), Postoperative (infections, bleeding); ^§§^ Outcomes: Successful (without the previous symptoms), Not-Resolutive (symptoms persisting). **Sex**: M = Male, F = Female; **Age:** Patient’s age in years; **Side**: L = Left side, R = Right side; **Cause**: T = Trauma, D = Deformity, Unknown = Unknown cause; **Complications**: NO = No complications, Intra-op = Intraoperative complication, Post-op = Postoperative complication; **Outcomes**: Resolved = Resolution of breathing issues, Not Resolved = Persistence of symptoms; **Operative Time**: Duration of the procedure in minutes.

**Table 2:** Endoscope group.

Patient	Sex	Age	Side *	Cause **	Complications ^§^	Outcomes ^§§^	Operative Time
1	M	44	L	T	NO	Resolutive	75′
2	M	36	R	T	NO	Resolutive	70′
3	M	37	R	T	NO	Resolutive	70′
4	W	39	L	T	NO	Resolutive	80′
5	M	27	L	T	NO	Resolutive	75′
6	W	55	R	Unknown	Post-op	Resolutive	80′
7	M	34	L	T	NO	Not Res.	70′
8	M	62	L	T	Intra-op	Resolutive	75′
9	W	42	R	D	NO	Resolutive	65′
10	M	26	R	Unknown	NO	Not Res.	70′
11	W	49	L	T	Post-op	Resolutive	75′
12	M	35	L	T	NO	Not Res.	80′
13	M	46	R	T	NO	Resolutive	70′
14	M	48	L	T	NO	Resolutive	85′
15	W	34	L	Unknown	Post-op	Resolutive	75′
16	M	53	L	T	NO	Not Res.	90′
17	M	31	R	T	NO	Resolutive	70′
18	W	27	L	T	NO	Resolutive	80′
19	M	38	L	Unknown	NO	Resolutive	90′
20	W	41	R	T	NO	Resolutive	65′
21	W	32	R	T	NO	Resolutive	75′
22	M	61	L	T	NO	Resolutive	80′
23	M	37	L	T	NO	Resolutive	85′
24	W	54	R	Unknown	NO	Resolutive	70′
25	M	28	L	T	NO	Resolutive	90′
26	W	47	L	T	NO	Resolutive	80′

* Side of deviation: L (Left), R (Right); ** Cause: T (post-Traumatic deviation), D (Drugs); ^§^ Complications: Intraoperative (bleeding), Postoperative (infections, bleeding); ^§§^ Outcomes: Successful (without the previous symptoms), Not-Resolutive (symptoms persisting). **Legend**: **Sex**: M = Male, F = Female; **Age**: Patient’s age in years; **Side**: L = Left side, R = Right side; **Cause**: T = Trauma, D = Deformity, Unknown = Unknown cause; Complications: NO = No complications, Intra-op = Intraoperative complication, Post-op = Postoperative complication; Outcomes: Resolved = Resolution of breathing issues, Not Resolved = Persistence of symptoms.

**Table 3:** Comparison between the endoscope-assisted and exoscope-assisted septoplasty procedures.

Aspect	Endoscope-Assisted Septoplasty	Exoscope-Assisted Septoplasty
Visualization	Direct visualization using endoscopic camera	3D high-definition magnification displayed on 4K monitor
Technique	Direct visualization using endoscopic camera	Instruments manipulated externally while observing 3D image
Surgical Field	Limited field of view	Wide, 3D stereoscopic view, enhancing depth perception
Ergonomics	Surgeon’s neck and back can become strained over time due to positioning	Improved posture and ergonomics; operator can adjust monitor freely
Training and Learning Curve	Learning curve based on traditional visual methods	Enhanced learning opportunities; trainees can view the surgical field
Time Efficiency	Average operative time of 70–90 min	Potentially reduced operative times with experience
Complications	Risk of intraoperative bleeding, especially in anterior regions	Slight increase in intraoperative bleeding during early use, but no long-term issues
Postoperative Outcomes	High success rate with minimal complications	Comparable success with less postoperative discomfort due to better visualization
Cost	Generally more affordable due to standard equipment	Exoscopic systems are more expensive and less accessible for some centers

## Data Availability

The original contributions presented in this study are included in the article. Further inquiries can be directed to the corresponding author.
